# Treatment of pruritus in a palliative care patient with low-dose paroxetine: a case report

**DOI:** 10.1186/s13256-017-1437-6

**Published:** 2017-10-02

**Authors:** Roni Y. Kraut

**Affiliations:** Department of Family Medicine, Faculty of Medicine and Dentistry, University of Alberta, Edmonton, AB Canada

**Keywords:** Pruritus, Palliative care, Paroxetine, Case report

## Abstract

**Background:**

Pruritus is a distressing symptom seen in palliative care. There is limited high-quality evidence of pharmaceutical treatments for pruritus in palliative care, including the use of paroxetine.

**Case presentation:**

I present a case of a 70-year-old caucasian woman with metastatic ovarian cancer who presented with severe pruritus. She had been diagnosed with bile duct obstruction 1 month earlier. Antihistamines and over-the-counter skin creams were first trialed, to no effect. Paroxetine was started at 5 mg in the evening, with the intention of titrating up. However, 5 mg of paroxetine was effective, and the patient’s pruritus fully resolved after the second day.

**Conclusions:**

This case supports the use of paroxetine as a therapy for pruritus in palliative care patients and suggests that paroxetine may be effective at a very low dose.

## Background

Pruritus is a distressing symptom seen in palliative care. It can be associated with any cancer, and it is more frequently seen in patients with hematologic, solid tumors (paraneoplasia) and biliary tract malignancies [1]. It also is associated with nonmalignant palliative cases, including chronic renal failure (uremia), biliary cirrhosis, and end-stage human immunodeficiency virus (HIV) [1].

The pathophysiology of pruritus is not fully understood, with many chemical mediators of pruritus having been identified, including histamine, serotonin, cytokines, opioids, and neuropeptides [2]. Further, the optimal pharmaceutical therapy in palliative care for pruritus secondary to malignancy is unclear; studies and guidelines provide contrary recommendations [3–5]. This is reflected in the conclusion of authors of a 2016 Cochrane systematic review which indicated that there was insufficient evidence to recommend a treatment of pruritus secondary to malignancy [1]. In this report, I present a case of a woman with severe pruritus secondary to metastatic ovarian adenocarcinoma.

## Case presentation

A 70-year-old white woman presented to my institution with severe pruritus. She had been diagnosed with stage IV adenocarcinoma of the ovaries with metastasis to the liver 1 year prior. She had undergone three rounds of carboplatin paclitaxel, followed by debulking surgery, followed by an additional six rounds of carboplatin paclitaxel therapy. After her last chemotherapy treatment, her carcinoembryonic antigen normalized. Four months later, she presented with jaundice, dark urine, and pale stools. A computed tomographic scan revealed the cancer had returned to the liver and was now obstructing her biliary duct. A stent was attempted several days later but was not successful. In collaboration with her oncologist, it was decided not to pursue further active treatment, and palliative care was initiated. She was otherwise healthy. The only medication she was taking was pantoprazole 40 mg daily for acid reflux. Her weight was 80 pounds.

One month later, she developed intense pruritus throughout her entire body. It was worse at night, and her sleep was significantly disturbed. She indicated her pruritus was 8/10 on a numerical rating scale (NRS), with a score of 0 reflecting no pruritus and a score of 10 reflecting the worst pruritus. She had no significant skin lesions or excoriations. She first tried over-the-counter moisturizers, with minimal effect. Benadryl was then suggested; it decreased her night waking, but she was too drowsy the following day. Loratadine was also attempted, to no effect. Cholestyramine was suggested, but she declined it due to the gastrointestinal side effects and longer time until onset of action.

The etiology of her pruritus appeared to be centrally induced, as opposed to prurioreceptive, neuropathic, or psychogenic [4]. My colleagues and I decided to try paroxetine on the basis of its preferable side effect profile, particularly in that it is less sedating. She was started on 5 mg nightly, with the intention to increase the dose as tolerated. The first night, her pruritus was 50% improved (NRS 4.5/10), and she was able to sleep better. The second night, her pruritus had fully resolved (NRS 0/10). She reported no nausea or vomiting. She was gratified with the effectiveness of paroxetine. She continued on 5 mg of paroxetine nightly, with no pruritus until she died 1 week later.

## Discussion

To the best of my knowledge, this is the first case study illustrating the effectiveness of 5 mg per day of paroxetine in an adult palliative care patient with pruritus. This is one-fourth the dose recommended for depression (20 mg per day) [6].

Authors of previously published case studies and case series have reported paroxetine to be effective at doses averaging 20 mg per day [7–12]. These series include a wide variety of palliative cases, including paraneoplastic syndrome (secondary to bronchus carcinoma, prostate carcinoma, and colon carcinoma) [7], opioid-induced pruritus [7], polycythemia vera [8–10], prostate cancer [11], and obstructive jaundice secondary to a primary malignancy (pancreatic cancer, rectal cancer, and cholangiocarcinoma) [13].

Only one randomized controlled trial on paroxetine for pruritus in palliative patients has been conducted: a double-blind, randomized, controlled crossover trial that took place at 2 hospices (in The Netherlands and Poland) with a total of 26 patients [13]. The dose of paroxetine was 20 mg per day, and the patients had a wide range of palliative conditions. The quality of the study was moderate due to the small sample size, undisclosed allocation concealment, and undisclosed method of randomization [1]. The study researchers found that mean pruritus during the last 3 days significantly decreased: NRS 6/10 in the placebo group versus 4.7/10 in the paroxetine group. The mean difference was 1.35 (SE 0.61–2.08). In addition, 9 of the 24 patients had their pruritus reduced by 50% or more. It is unclear if this subset of patients shared similar characteristics.

Nausea and vomiting have been common side effects of paroxetine in palliative care patients [7, 13]. They are often transient and occur on initiation of paroxetine. In the randomized controlled trial, 2 of the 26 patients were withdrawn from the study due to severe nausea and vomiting [13]. Other side effects that have been reported include fatigue [7, 8, 11, 13] and sexual dysfunction [8]. This is consistent with side effects of paroxetine for depression, with nausea and fatigue being the more frequent side effects, at 25% and 23%, respectively [6]. It has been suggested that paroxetine be started at a low dose and titrated up to decrease the occurrence of nausea and vomiting [3, 5, 13].

## Conclusions

Among the myriad of pharmaceutical options available to clinicians and the lack of a known optimal treatment, this case provides more evidence that paroxetine may be effective for pruritus in palliative care patients with malignancies. It further reinforces that paroxetine should be started at a low dose and titrated up to achieve effectiveness.
